# Supplementary material for: What is the impact of structural changes in society on diabetes self-management and trajectories of HbA1c? A cohort study before, during and after the COVID-19 pandemic in people with diabetes treated at outpatient clinics
Source: PLoS One. 2025 Aug 12;20(8):e0329394. doi: 10.1371/journal.pone.0329394 (PMC12342243; doi:10.1371/journal.pone.0329394)
Supplement: S1 Appendix — The questionnaire used to collect data on diabetes self-management and personal characteristics. The questionnaire is in Danish. (DOCX) [file pone.0329394.s001.docx]

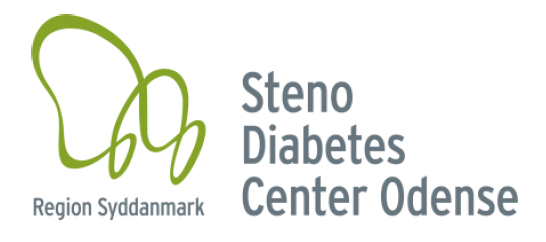


**Spørgeskemaundersøgelse: Trivsel og tilfredshed blandt diabetespatienter i Region Syddanmark**

**PATIENTTILFREDSHED UNDER CORONAPANDEMIEN**

**I perioden fra marts måned 2020 til marts måned 2021**

|  | Ja | Nej | Ved ikke |  |
| --- | --- | --- | --- | --- |
| Har du været smittet med Coronavirus? | ❑ | ❑ |  |  |

|  | Ja, (påbegyndt eller afsluttet) | Nej |  |
| --- | --- | --- | --- |
| Er du vaccineret mod Coronavirus? | ❑ | ❑ |  |

|  | Ja | Nej | Ikke relevant |
| --- | --- | --- | --- |
| Har du været hjemsendt fra dit arbejde eller din uddannelse under Coronapandemien? | ❑ | ❑ | ❑ |

|  |  |  |  |
| --- | --- | --- | --- |
| Angiv cirka antal måneder for hjemsendelse: |  | _ _ _ _ |  |

**ÆNDRINGER I DIN SUNDHED OG HVERDAGEN UNDER CORONAPANDEMIEN**

5.1 Hvordan har Coronapandemien påvirket din nuværende håndtering af diabetes i hverdagen?

|  | Betydeligt sværere at håndtere (1) | Noget sværere at håndtere (2) | Lidt sværere at håndtere  (3) | Ingen påvirkning  (4) | Lidt lettere at håndtere  (5) | Noget lettere at håndtere  (6) | Betydeligt lettere at håndtere  (7) |
| --- | --- | --- | --- | --- | --- | --- | --- |
|  |  |  |  |  |  |  |  |

5.2 Sammenlignet med før Coronapandemien, har mængden af mad, som du spiser, ændret sig?

|  | Spiser meget mere  (1) | Spiser noget mere  (2) | Spiser lidt mere  (3) | Spiser omtrent den samme mængde (4) | Spiser lidt mindre  (5) | Spiser noget mindre  (6) | Spiser meget  mindre  (7) |
| --- | --- | --- | --- | --- | --- | --- | --- |
|  |  |  |  |  |  |  |  |

5.3 Sammenlignet med før Coronapandemien, hvordan vil du beskrive din nuværende kost i forhold til din diabetes?

|  | Meget mindre sund  (1) | Noget mindre sund  (2) | Lidt mindre sund  (3) | Ingen ændring    (4) | Lidt sundere    (5) | Noget sundere  (6) | Meget sundere  (7) |
| --- | --- | --- | --- | --- | --- | --- | --- |
|  |  |  |  |  |  |  |  |

5.4 Sammenlignet med før Coronapandemien, hvor fysisk aktiv er du?

|  | Meget mindre  (1) | Noget mindre  (2) | Lidt mindre  (3) | Ingen ændring  (4) | Lidt mere    (5) | Noget mere  (6) | Meget mere  (7) |
| --- | --- | --- | --- | --- | --- | --- | --- |
|  |  |  |  |  |  |  |  |

5.5 Sammenlignet med før Coronapandemien, hvordan har din vægt ændret sig?

|  | Meget lavere  (1) | Noget lavere  (2) | Lidt lavere  (3) | Ingen ændring  (4) | Lidt højere    (5) | Noget højere  (6) | Meget højere  (7) |
| --- | --- | --- | --- | --- | --- | --- | --- |
|  |  |  |  |  |  |  |  |

5.6 Sammenlignet med før Coronapandemien, hvordan vil du beskrive din nuværende brug af diabetes-medicin?

|  | Jeg tager min diabetes-medicin meget mere regelmæssigt (1) | Jeg tager min diabetes-medicin lidt mere regelmæssigt (2) | Ingen ændring  (3) | Jeg tager min diabetes-medicin lidt mindre regelmæssigt (4) | Jeg tager min diabetes-medicin meget mindre regelmæssigt (5) |
| --- | --- | --- | --- | --- | --- |
|  |  |  |  |  |  |

**5.7 Sammenlignet med før Coronapandemien, hvor ofte tjekker du dit blodsukker?**

|  | Sjældnere (1) | Cirka lige så ofte (2) | Oftere (3) |
| --- | --- | --- | --- |
| Tjekker blodsukker med teststrimler eller tjekker din CGM: |  |  |  |

| Skriv venligst her, hvis du har kommentarer til ændringer i din sundhed under Coronapandemien: |
| --- |
|  |

**MENTAL TRIVSEL OG SYGDOMSHÅNDTERING**

**Ved hvert af de 5 udsagn, sæt venligst et kryds i det felt der kommer tættest på, hvordan du har haft det i de sidste to uger.**

|  | Hele  tiden | Det meste af tiden | Lidt mere end halvdelen af tiden | Lidt mindre end halvdelen af tiden | Lidt af tiden | På intet tidspunkt |
| --- | --- | --- | --- | --- | --- | --- |
| I de sidste 2 uger… |  |  |  |  |  |  |
| … har jeg været glad og i godt humør | ❑ | ❑ | ❑ | ❑ | ❑ | ❑ |
| … har jeg følt mig rolig og afslappet | ❑ | ❑ | ❑ | ❑ | ❑ | ❑ |
| … har jeg følt mig aktiv og energisk | ❑ | ❑ | ❑ | ❑ | ❑ | ❑ |
| … er jeg vågnet frisk og udhvilet | ❑ | ❑ | ❑ | ❑ | ❑ | ❑ |
| … har min dagligdag været fyldt med ting der interesserer mig | ❑ | ❑ | ❑ | ❑ | ❑ | ❑ |

**Hvilke af følgende diabetesforhold er for tiden et problem for dig?
Sæt venligst ét kryds i hver linje i det felt, der bedst dækker dit svar.**

|  | Ikke et problem | Mindre problem | Moderat problem | Forholdsvis alvorligt problem | Alvorligt problem |
| --- | --- | --- | --- | --- | --- |
| At du føler dig skræmt ved tanken om at leve med diabetes? | ❑ | ❑ | ❑ | ❑ | ❑ |
| At du føler dig nedtrykt ved tanken om at leve med diabetes? | ❑ | ❑ | ❑ | ❑ | ❑ |
| At du er bekymret for fremtiden og risikoen for alvorlige komplikationer? | ❑ | ❑ | ❑ | ❑ | ❑ |
| At du føler, at din diabetes tager for meget at din energi mentalt og fysisk i hverdagen? | ❑ | ❑ | ❑ | ❑ | ❑ |
| At kunne magte komplikationer til diabetes? | ❑ | ❑ | ❑ | ❑ | ❑ |

**De næste spørgsmål handler om, hvordan du håndterer din diabetes**

**Du bedes sætte ét kryds i hver linje ved det svar, der bedst passer til din grad af enighed ud fra følgende svarmuligheder (Besvar venligst alle spørgsmål)**

|  | Meget uenig | Noget uenig | Lidt uenig | Neutral | Lidt enig | Noget enig | Meget enig |
| --- | --- | --- | --- | --- | --- | --- | --- |
| Jeg føler mig tryg ved min evne til at klare min diabetes |  |  |  |  |  |  |  |
| Jeg føler mig i stand til at håndtere min diabetes nu |  |  |  |  |  |  |  |
| Jeg er i stand til at varetage den rutinemæssige pasning af min diabetes |  |  |  |  |  |  |  |
| Jeg er i stand til at møde den udfordring, det er at styre min diabetes |  |  |  |  |  |  |  |

**BAGGRUNDSOPLYSNINGER**

|  | Mand | Kvinde | Ønsker ikke at oplyse |
| --- | --- | --- | --- |
| Hvad er dit køn? | ❑ | ❑ | ❑ |

|  |  |  | Ønsker ikke at oplyse |
| --- | --- | --- | --- |
| Hvornår er du født?  Skriv årstal (ex 1984) |  | _ _ _ _ | ❑ |

|  | Type 1 diabetes | Type 2 diabetes | Anden diabetes |
| --- | --- | --- | --- |
| Hvilken type diabetes har du? | ❑ | ❑ | ❑ |

|  |  |  |  |
| --- | --- | --- | --- |
| Hvilket år blev du diagnosticeret med diabetes?  Skriv årstal (ex 1984) |  | _ _ _ _ |  |

|  |  |
| --- | --- |
| Har du en eller flere af følgende diabeteskomplikationer? (det er muligt at sætte flere krydser) | |
| ❑ | Øjensygdom (retinopati) |
| ❑ | Nyresygdom (nefropati) |
| ❑ | Nervebetændelse pga. diabetes (diabetisk neuropati) |
| ❑ | Fodsår |
| ❑ | Hjertekarsygdom |

|  | Enlig | Samlevende | Gift | Andet | Ønsker ikke at oplyse |
| --- | --- | --- | --- | --- | --- |
| Hvad er din civilstatus? | ❑ | ❑ | ❑ | ❑ | ❑ |

| **Hvad er din højst fuldførte uddannelse?** |
| --- |
| - Grundskolen (1.-10. klasse) |
| - Gymnasial uddannelse (f.eks. gymnasiet, HF, HTX) |
| - Erhvervsuddannelse (f.eks. håndværker, handel og kontor, frisør, social- og sundhedsassistent) |
| - Kort videregående uddannelse (f.eks. økonoma, maskintekniker, tandplejer) - Mellemlang videregående uddannelse (f.eks. professionsbachelor, lærer, sygeplejerske) |
| - Lang videregående uddannelse og forskeruddannelse (universitetsuddannelse, f.eks. læge, gymnasielærer, jurist, ingeniør, ph.d.) |
| - Andet |
| - Ønsker ikke at oplyse |
