# Supplementary material for: What is the impact of structural changes in society on diabetes self-management and trajectories of HbA1c? A cohort study before, during and after the COVID-19 pandemic in people with diabetes treated at outpatient clinics
Source: PLoS One. 2025 Aug 12;20(8):e0329394. doi: 10.1371/journal.pone.0329394 (PMC12342243; doi:10.1371/journal.pone.0329394)
Supplement: S2 Appendix — Supplementary analyses include descriptive statistics and analyses according to diabetes type. (DOCX) [file pone.0329394.s002.docx]

Content

[Other diabetes types 2](#_Toc187756567)

[Imputation output 4](#_Toc187756568)

[Crude analyses (models only adjusted for age and sex) 5](#_Toc187756569)

[Sensitivity analyses 6](#_Toc187756570)

[Different not placements 6](#_Toc187756571)

[Restricted to those with at least one HbA1c measurement per year 7](#_Toc187756572)

[Restricted to those with at least one Hba1c measurement during early Covid-19 (between the 11^th^ of March and the 31^st^ of July) as well as at least one measurement before and one measurement after 8](#_Toc187756573)

[Analyses stratified by diabetes type 9](#_Toc187756574)

[Type 1-diabetes 9](#_Toc187756575)

[Type 2-diabetes 10](#_Toc187756576)

[Assessment of construction validity 11](#_Toc187756577)

[Contingency table: Change in bodyweight and change in diet 11](#_Toc187756578)

[Contingency table: Change in bodyweight and change in physical activity 11](#_Toc187756579)

# Other diabetes types

|  | **Other** |
| --- | --- |
| **N** | 251 |
| **Age (years), median (q1-q3)** | 61 (53-69) |
| **Men (n (%))** | 130 (51.8 %) |
| **Cohabitation status** |  |
| Partner or married | 165 (74.0 %) |
| Single | 52 (23.3 %) |
| Other/not declared | 6 ( 2.7 %) |
| Missing | 28 |
| **Educational attainment** |  |
| Elementary | 29 (11.6 %) |
| High school or vocational | 94 (37.8 %) |
| Short or bachelor level | 86 (34.5 %) |
| Masters degree or higher | 21 ( 8.4 %) |
| Other/not declared | 19 ( 7.6 %) |
| Missing | 2 |
| **Diabetes duration (years), median (q1-q3)** | 12 (4-20) |
| Missing | 2 |
| **Number of complications** |  |
| 0 | 184 (73.3 %) |
| 1 compl. | 42 (16.7 %) |
| 2 compl. | 17 ( 6.8 %) |
| 3 or more compl. | 8 ( 3.2 %) |
| **Has been infected with the coronavirus** |  |
| No/do not know | 233 (95.1 %) |
| Yes | 12 ( 4.9 %) |
| N missing | 6 |
| **Vaccinated against the coronavirus** |  |
| No | 131 (53.5 %) |
| Yes | 114 (46.5 %) |
| N missing | 6 |
| **Sent home due to the coronavirus** |  |
| No/not relevant | 186 (76.2 %) |
| Yes | 58 (23.8 %) |
| Months sent home due to the coronavirus (months), median (q1-q3) | 3 (2-6) |
| N missing | 7 |
| **PAID-5 (score 0-20), median (q1-q3)** | 6 (2-10) |
| N missing | 12 |
| **WHO-5 (score 0-100), median (q1-q3)** | 60 (44-80) |
| N missing | 12 |

Trajectories of HbA1c before and during COVID-19 for people with other diabetes types


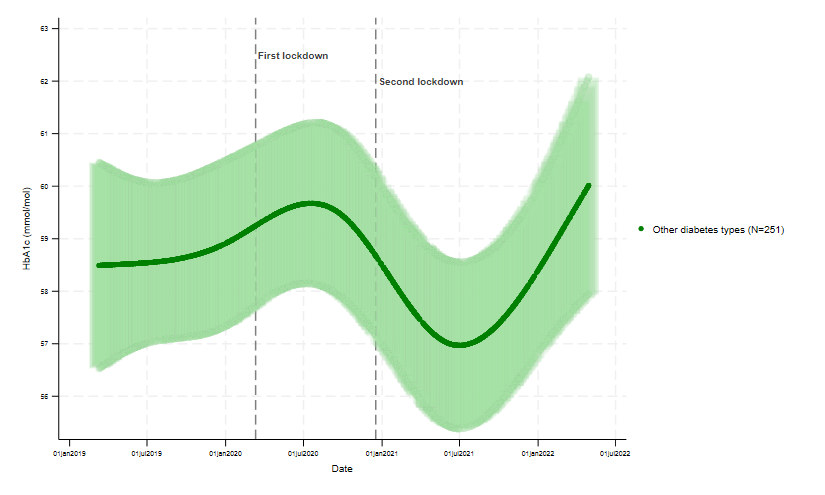


# Imputation output

We created 10 datasets of imputed data

| **Variable** | **Imputation model** | **Complete** | **Incomplete** | **Imputed** | **Total** |
| --- | --- | --- | --- | --- | --- |
| Change in diet | Ordinal logistic regression | 4,939 | 237 | 237 | 5,176 |
| Change in physical activity | Ordinal logistic regression | 5,003 | 173 | 173 | 5,176 |
| Change in regularity of medication intake | Ordinal logistic regression | 5,026 | 150 | 150 | 5,176 |
| Relationship status | Ordinal logistic regression | 4,688 | 488 | 488 | 5,176 |
| Educational attainment | Ordinal logistic regression | 5,116 | 60 | 60 | 5,176 |
| Age at diabetes onset (used to calculate diabetes duration) | Predictive mean matching | 4,915 | 261 | 261 | 5,176 |

#
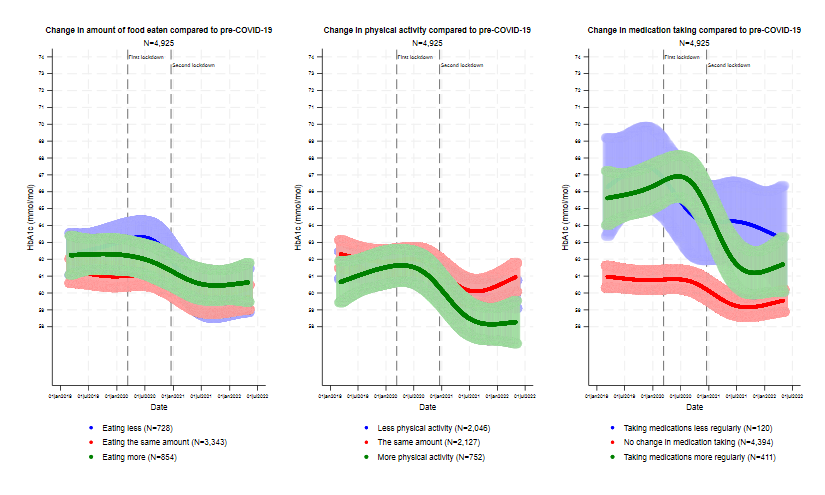
Crude analyses (models only adjusted for age and sex)

# Sensitivity analyses

##
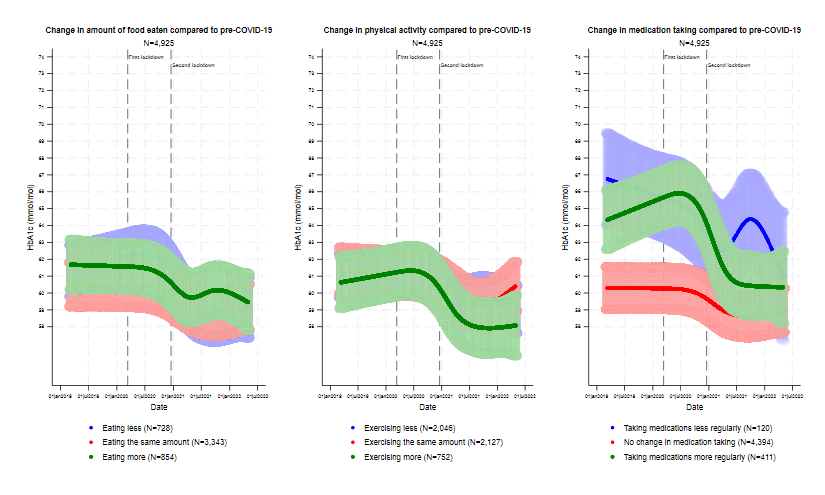
Different not placements

*Adjusted for age (yrs), sex (male/female), diabetes duration (yrs), number of diabetes-related complications (0, 1, 2, ≥3) and educational attainment (elementary, high school or vocational, short or bachelor level, master’s degree or higher, other/not declared). The curves are estimated for a 50-year-old female who has had type 1-diabetes for 15 years, who has two diabetes-related complications and a short or bachelor level educational attainment. The frequencies within each category of the change variables is determined based on the data in the first imputed dataset as an example.*

## Restricted to those with at least one HbA1c measurement per year


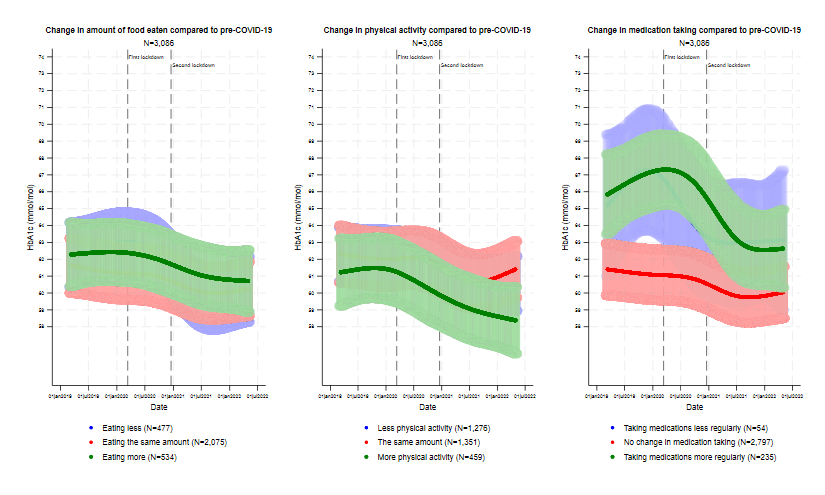


*Adjusted for age (yrs), sex (male/female), diabetes duration (yrs), number of diabetes-related complications (0, 1, 2, ≥3) and educational attainment (elementary, high school or vocational, short or bachelor level, master’s degree or higher, other/not declared). The curves are estimated for a 50-year-old female who has had type 1-diabetes for 15 years, who has two diabetes-related complications and a short or bachelor level educational attainment. The frequencies within each category of the change variables is determined based on the data in the first imputed dataset as an example.*

## Restricted to those with at least one Hba1c measurement during early Covid-19 (between the 11^th^ of March and the 31^st^ of July) as well as at least one measurement before and one measurement after


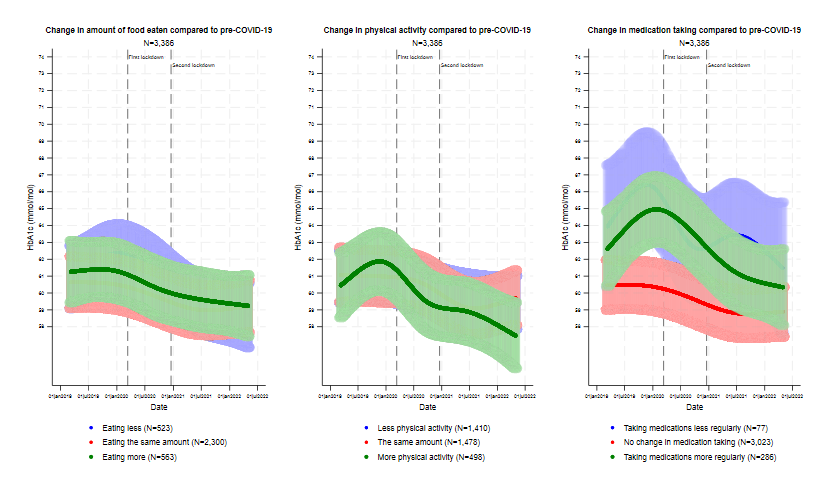


*Adjusted for age (yrs), sex (male/female), diabetes duration (yrs), number of diabetes-related complications (0, 1, 2, ≥3) and educational attainment (elementary, high school or vocational, short or bachelor level, master’s degree or higher, other/not declared). The curves are estimated for a 50-year-old female who has had type 1-diabetes for 15 years, who has two diabetes-related complications and a short or bachelor level educational attainment. The frequencies within each category of the change variables is determined based on the data in the first imputed dataset as an example.*

# Analyses stratified by diabetes type

##
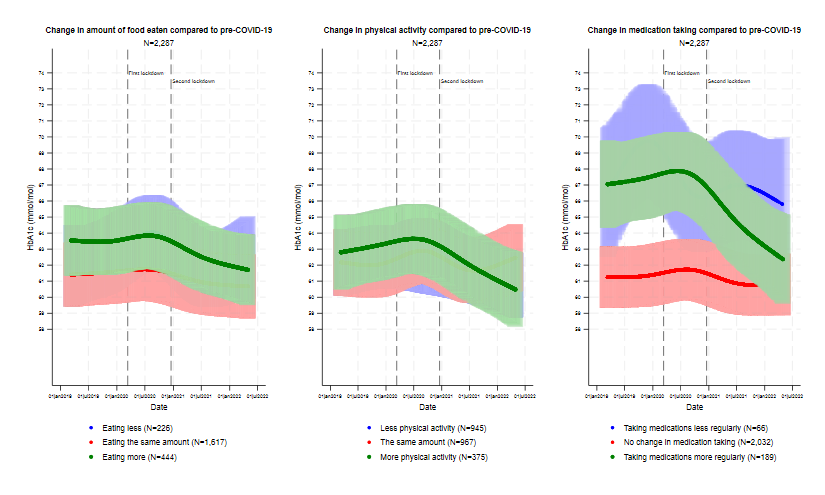
Type 1-diabetes

*Adjusted for age (yrs), sex (male/female), diabetes duration (yrs), number of diabetes-related complications (0, 1, 2, ≥3) and educational attainment (elementary, high school or vocational, short or bachelor level, master’s degree or higher, other/not declared). The curves are estimated for a 50-year-old female who has had type 1-diabetes for 15 years, who has two diabetes-related complications and a short or bachelor level educational attainment. The frequencies within each category of the change variables is determined based on the data in the first imputed dataset as an example.*

##
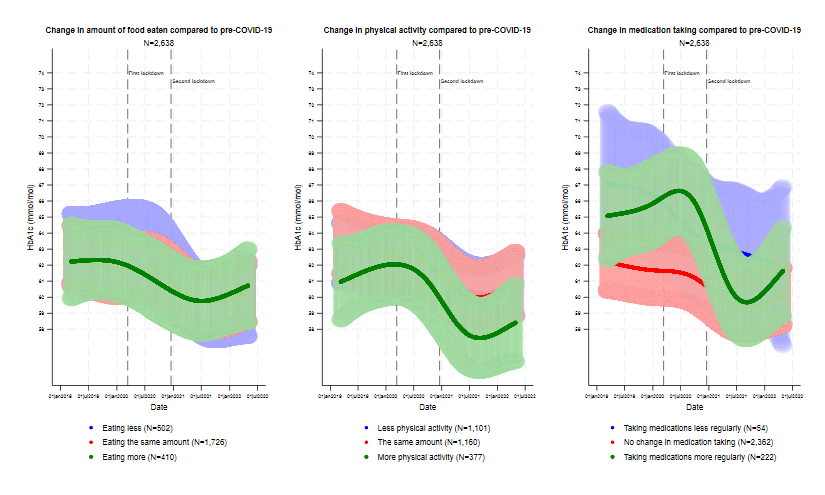
Type 2-diabetes

*Adjusted for age (yrs), sex (male/female), diabetes duration (yrs), number of diabetes-related complications (0, 1, 2, ≥3) and educational attainment (elementary, high school or vocational, short or bachelor level, master’s degree or higher, other/not declared). The curves are estimated for a 50-year-old female who has had type 2-diabetes for 15 years, who has two diabetes-related complications and a short or bachelor level educational attainment. The frequencies within each category of the change variables is determined based on the data in the first imputed dataset as an example.*

# Assessment of construction validity

## Contingency table: Change in bodyweight and change in diet

|  | **Eating less** | **Eating the same amount** | **Eating more** |
| --- | --- | --- | --- |
| **Increased bodyweight, n (%)** | 103 (13.6) | 780 (22.2) | 602 (66.7) |
| **No change in bodyweight, n (%)** | 201 (26.5) | 2,164 (61.6) | 202 (22.4) |
| **Decreased bodyweight, n (%)** | 455 (60.0) | 570 (16.2) | 99 (11.0) |

## Contingency table: Change in bodyweight and change in physical activity

|  | **Less physical activity** | **The same amount** | **More physical activity** |
| --- | --- | --- | --- |
| **Increased bodyweight, n (%)** | 961 (44.6) | 366 (16.4) | 158 (20.0) |
| **No change in bodyweight, n (%)** | 775 (36.0) | 1,454 (65.2) | 338 (42.7) |
| **Decreased bodyweight, n (%)** | 417 (19.4) | 411 (18.4) | 296 (37.4) |
